# Supplementary material for: Characterization of a spectrally diverse set of fluorescent proteins as FRET acceptors for mTurquoise2
Source: Sci Rep. 2017 Sep 20;7:11999. doi: 10.1038/s41598-017-12212-x (PMC5607329; doi:10.1038/s41598-017-12212-x)
Supplement: Supplementary file 3 — Supplementary figures [file 41598_2017_12212_MOESM3_ESM.pdf]

Supplementary Information

Characterization of a spectrally diverse set of fluorescent proteins as FRET acceptors for mTurquoise2

Marieke Mastop*, Daphne S. Bindels*, Nathan C. Shaner#, Marten Postma*, Theodorus W.J. Gadella Jr.*, Joachim Goedhart*, ‡

* Swammerdam Institute for Life Sciences, Section of Molecular Cytology, van Leeuwenhoek Centre for Advanced Microscopy, University of Amsterdam, P.O. Box 94215, NL-1090 GE Amsterdam, The Netherlands.
#Department of Photobiology and Bioimaging, The Scintillon Institute, San Diego, California, United States of America

‡Correspondence to j.goedhart@uva.nl

Supplementary Figures

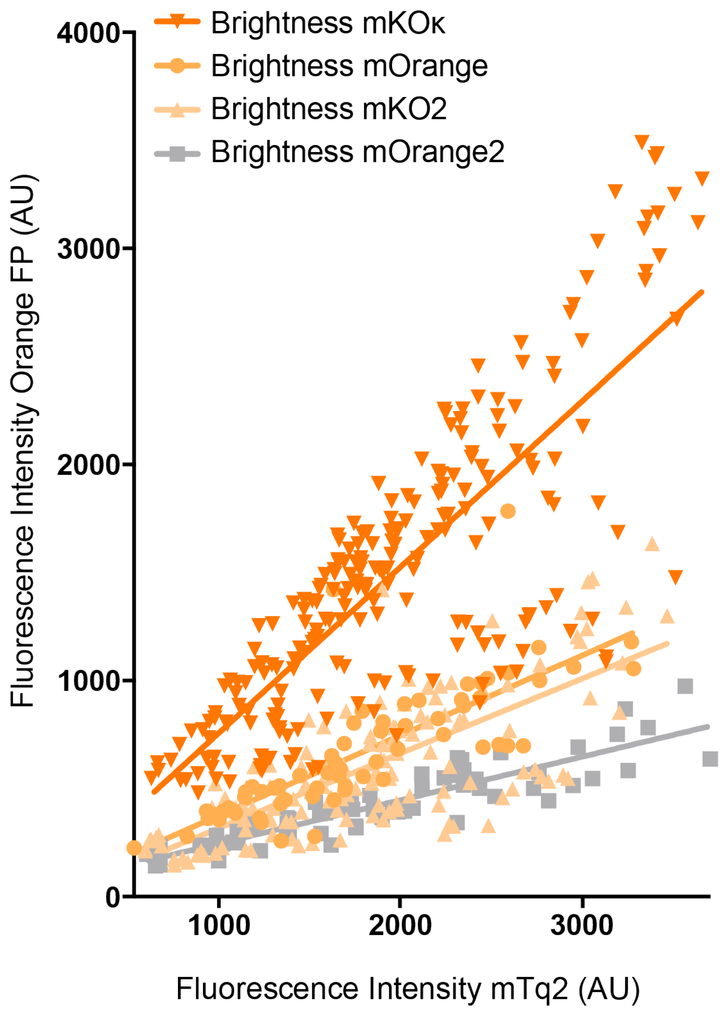

Figure S1. The brightness of orange fluorescent proteins (FPs).
The brightness of the orange FPs relative to mTurquoise2 by employing a T2A construct with both FPs resulting in the separate expression of both fluorescent proteins in equal amounts. Cells were imaged on a widefield fluorescence microscope (Axiovert 200 M; Carl Zeiss GmbH) equipped with a xenon arc lamp with monochromator (Cairn Research, Faversham, Kent, UK), using a 40x objective (oil-immersion Plan-Neo-fluor 40×/1.30; Carl Zeiss GmbH). Orange FPs were excited with 510nm light and emission was detected with a BP572/25 filter. As reference, mTurquoise2 was excited with 420nm light and emission was detected with a BP470/30 filter. After subtraction of background signal, the mean fluorescence intensity of the cells was calculated. Each dot represents a cell. The lines show the fit with a linear regression, the steeper the line the higher the brightness. The number of cells analyzed are: mOrange-T2A-mTq2 *n*=65, mOrange2-T2A-mTq2 *n*=67, mKO2-T2A-mTq2 *n*=147 and mKOκ-T2A-mTq2 *n*=239.

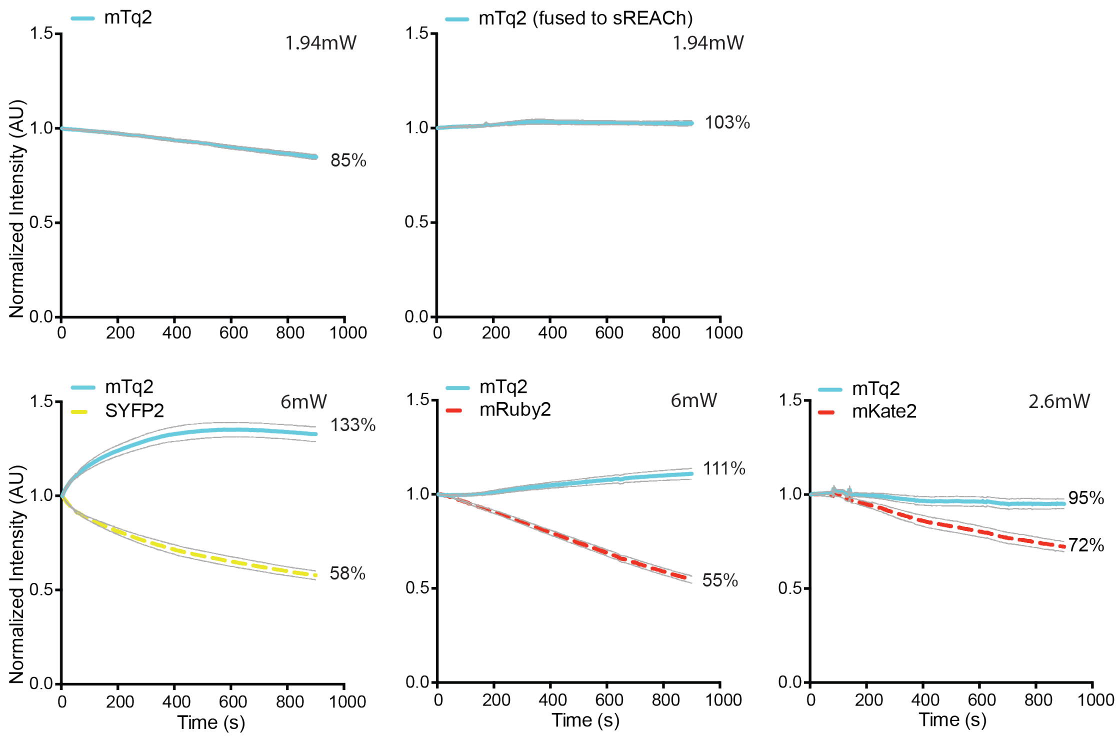

Figure S2. Photostability under FRET conditions.
Fusion constructs of mTurquoise2 and acceptor FP were used in this experiment. As control a construct only expressing mTurquoise2 is measured (upper left). The power is shown in the graphs. The error is indicated with grey lines and corresponds to 95% confidence interval. The photostability of the fusion constructs is shown under continuous illumination with 420nm light for 900s. Each 4s, fluorescence intensity of FRET donor and acceptor was recorded with an exposure time of 200ms using a 40x objective (oil-immersion Plan-Neo- fluor 40×/1.30; Carl Zeiss GmbH). mTurquoise2 emission was detected with a BP470/30 filter, GFP/YFP emission was detected with a BP535/30 filter and OFP/RFP emission was detected with a BP620/60 filter. After subtraction of background signal, the mean fluorescence intensity of the cells was calculated for each time point using ImageJ. The initial fluorescence intensity was set on 100% and it is stated what percentage of the initial fluorescence is left after 900s illumination. The number of cells imaged is: mTq2 n=18; sREACh-mTq2 n=12; SYFP2-mTq2 n=25; mRuby2-mTq2 n=36; mKate2-mTq2 n=18.

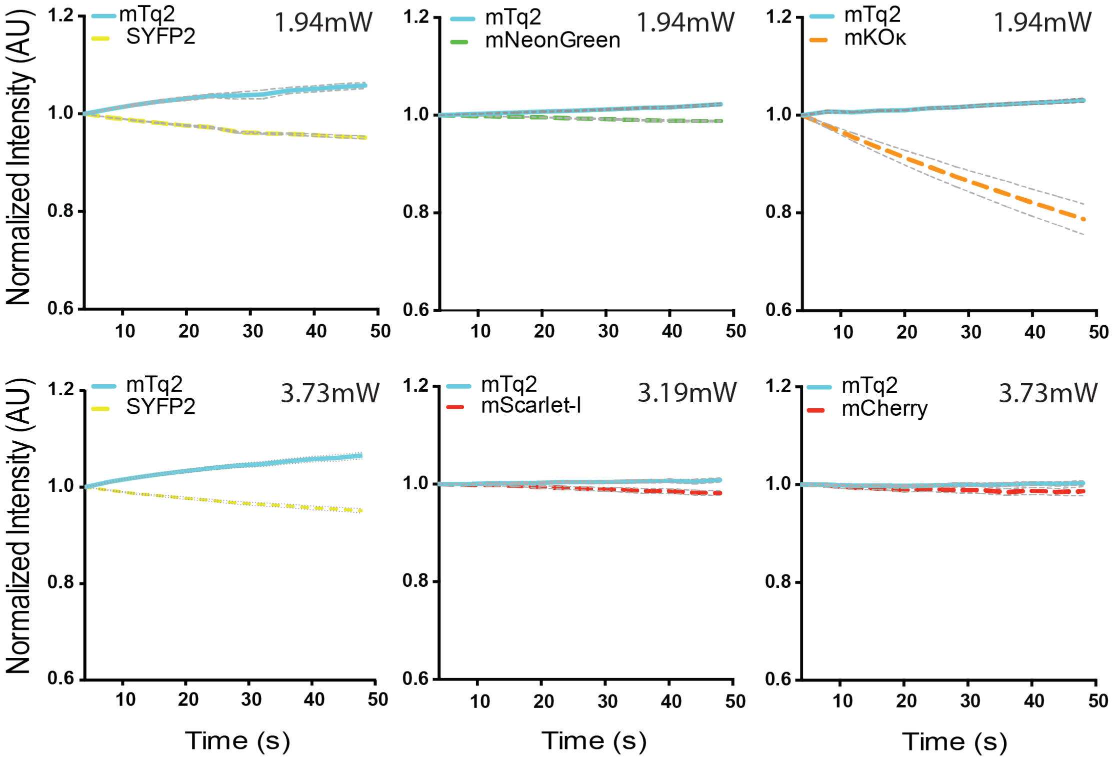

Figure S3. Photostability under FRET conditions for a duration that equals a typical FRET experiment.

This experiment shows in more detail the bleaching in the first 48s of the experiments shown in figure 5 and supplemental figure S2. Fusion constructs of mTurquoise2 and acceptor FP were used in this experiment. The power is shown in the graphs. The error is indicated with grey lines and corresponds to 95% confidence interval. The photostability of the fusion constructs is shown under continuous illumination with 420nm light for 48s. Each 4s, fluorescence intensity of FRET donor and acceptor was recorded with an exposure time of 200ms using a 40x objective (oil-immersion Plan-Neo- fluor 40×/1.30; Carl Zeiss GmbH). mTurquoise2 emission was detected with a BP470/30 filter, GFP/YFP emission was detected with a BP535/30 filter and OFP/RFP emission was detected with a BP620/60 filter. After subtraction of background signal, the mean fluorescence intensity of the cells was calculated for each time point using ImageJ. The initial fluorescence intensity was set on 100%. The number of cells imaged is: SYFP2-mTq2 *n*=23; mNeonGreen-mTq2 *n*=21; mKOκ-mTq2 *n*=15; SYFP2-mTq2 (3.73mW) *n*=23; mScarlet-I-mTq2 *n*=11; mCherry-mTq2 *n*=15.

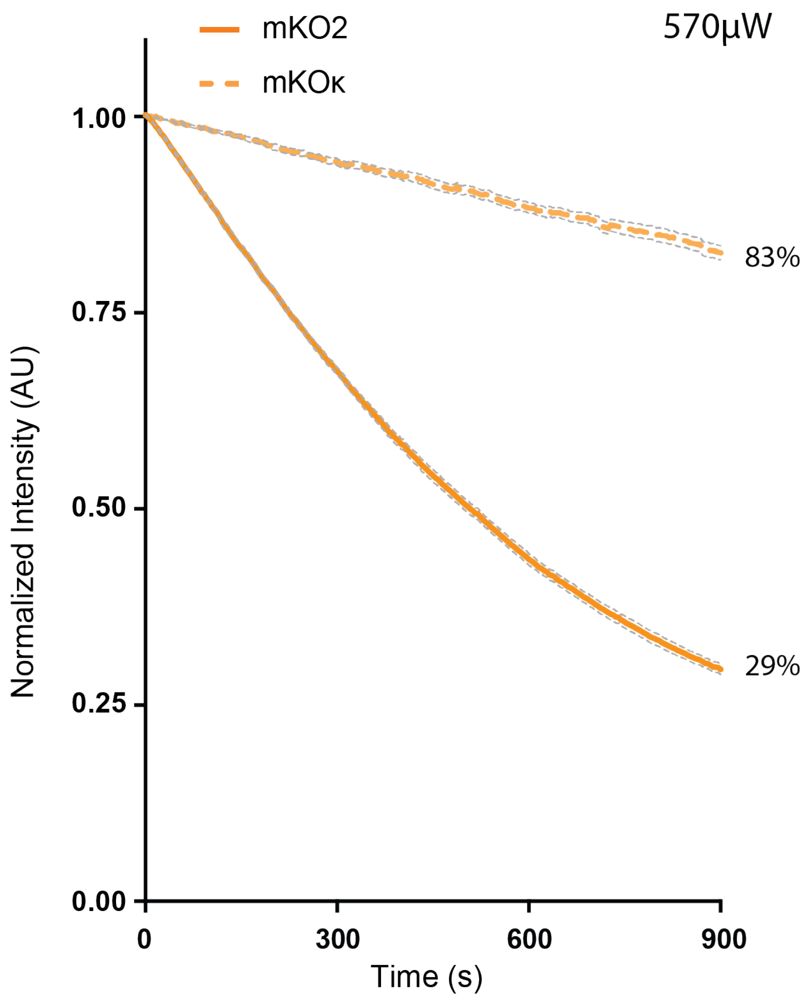

Figure S4. Photostability of mKO2 and mKOκ.
The graph shows the photostability of mKO2 and mKOκ, directly excited with 570nm light at a power of 570μW. Each 4s, fluorescence intensity of the orange fluorescent protein was recorded with an exposure time of 200ms using a 40x objective (oil-immersion Plan-Neo- fluor 40×/1.30; Carl Zeiss GmbH). The emission was detected with a BP620/60 filter. After subtraction of background signal, the mean fluorescence intensity of the cells was calculated for each time point using ImageJ. The initial fluorescence intensity was set on 100% and it is stated what percentage of the initial fluorescence is left after 900s continuous illumination. The error is indicated with grey lines and corresponds to 95% confidence interval. The number of cells imaged is: mKO2 *n*=7; mKOκ *n*=9.

Photobleaching of SYFP2 and SYFP2-145L

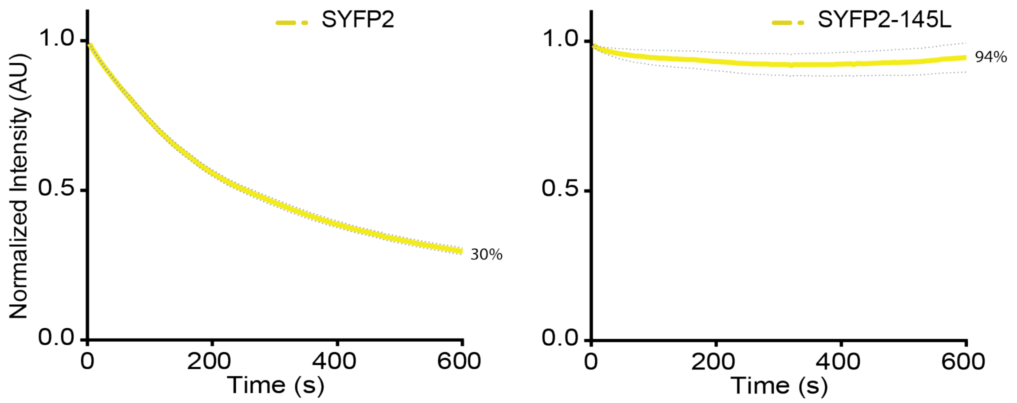

Fluoresence lifetime imaging of FRET pairs comparing SYFP2 and SYFP2-145L as FRET acceptor with donor mTurquoise2

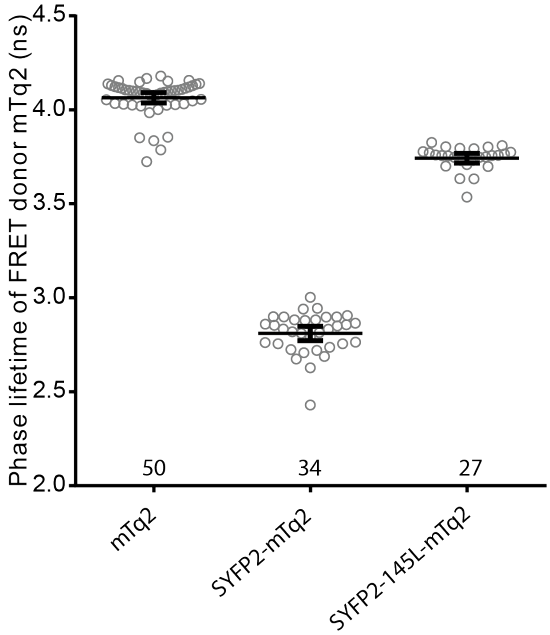

Figure S5. Photobleaching and FLIM-FRET of SYFP2 compared to SYFP2-145L.
The upper graphs show the photobleaching of HeLa cells expressing yellow fluorescent protein variants SYFP2 or SYFP2-145L, under continuous illumination for 600s with 500nm light at a power of 1.39mW. Cells were imaged on a widefield fluorescence microscope (Axiovert 200 M; Carl Zeiss GmbH) equipped with a xenon arc lamp with monochromator (Cairn Research, Faversham, Kent, UK), using a 40x objective (oil-immersion Plan-Neo- fluor 40×/1.30; Carl Zeiss GmbH). Each 4s, fluorescence intensity of the yellow fluorescent protein was recorded with an exposure time of 200ms using a 40x objective (oil-immersion Plan-Neo- fluor 40×/1.30; Carl Zeiss GmbH). The emission was recorded with a BP535/30 filter. The initial fluorescence intensity was set on 100% and it is stated what percentage of the initial fluorescence is left after 600s continuous illumination. The error bars display the 95% confidence interval. The number of cells imaged is: SYFP2 *n*=19; SYFP2-145L *n*=29.
In the lower graph the phase lifetime of mTurquoise2 (mTq2) when paired with either SYFP2 or SYFP2-145L as acceptor is depicted. Cells were imaged with a home-build instrument (Van Munster and Gadella, 2004) using a RF-modulated AOM and a RF-modulated image intensifier (Lambert Instruments II18MD) coupled to a CCD camera (Photometrics HQ) as detector, using a 40x objective (Plan NeoFluar NA 1.3 oil). The modulation frequency was set to 75.1MHz. At least twelve phase images with an exposure time of 20–100ms seconds were acquired in a random recording order (van Munster and Gadella Jr., 2004). As a reference the lifetime of untagged mTurquoise2 is shown. The dots indicate individual cells and the error bars show 95% confidence intervals. The number of cells imaged is mTq2 *n*=50; SYFP2-mTq2 *n*=34; SYFP2-145L-mTq2 *n*=27.

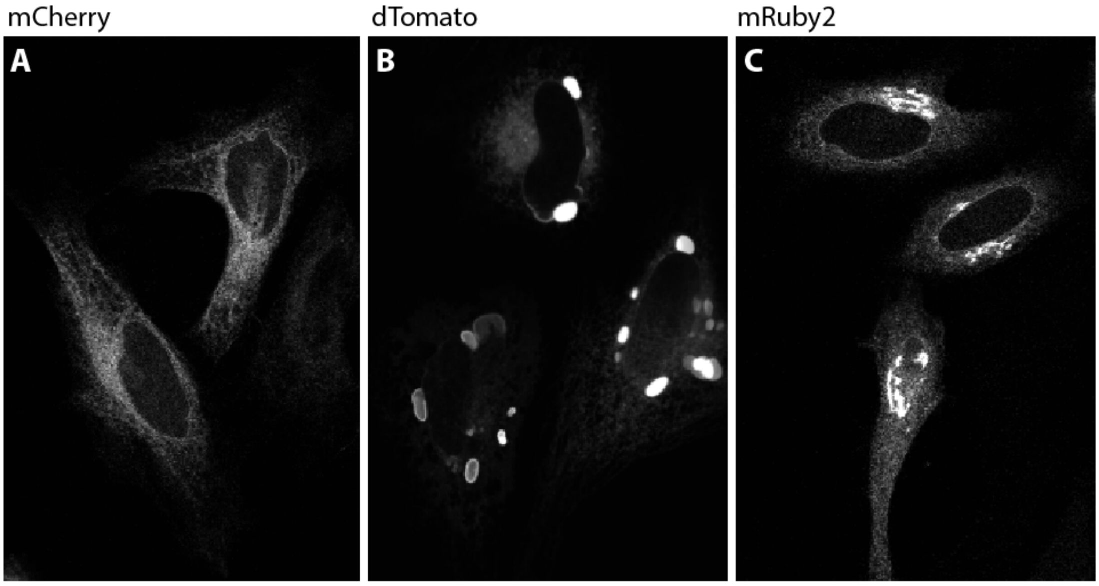

Figure S6. mRuby2 shows abnormal localization in the OSER assay.
This graph shows CytErm-FP fusion constructs that are used in the OSER assay to determine the dimerization tendency of an FP. Cells were imaged with a Nikon A1 confocal microscope equipped with a 60x oil immersion objective (Plan Apochromat VC, NA 1.4), with the pinhole size set at 1 Airy Unit. The FPs were excited with a 561nm laser and a BP595/50 emission filter was used. A monomeric FP such as mCherry (A) shows ER localization. If a FP has a high dimerization tendency, ER membranes fuse and form OSER structures, which is shown here for dTomato (B). It was impossible to perform this assay on mRuby2 since it showed what seemed Golgi localization instead of the expected ER and it is unknown what causes this abnormal localization. The width of the images is: 140μm.

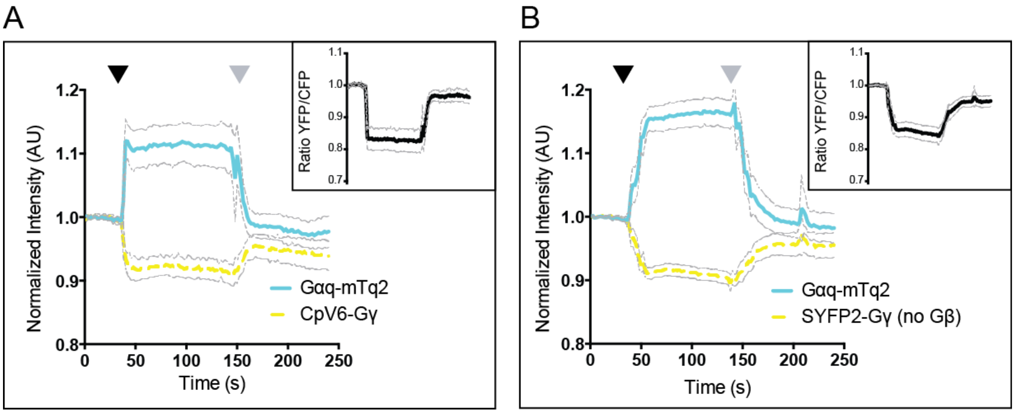

Figure S7. Comparison of a Gq activation biosensor with and without the Gβ subunit.
Hela cells over-expressing the histamine1 receptor and Gqsensor were stimulated with 100μM histamine after 42s (black arrow) and antagonized with 10μM pyrilamine after 146s (grey arrow). Cells were imaged on a widefield fluorescence microscope (Axiovert 200 M; Carl Zeiss GmbH) equipped with a xenon arc lamp with monochromator (Cairn Research, Faversham, Kent, UK), using a 40x objective (oil-immersion Plan-Neo- fluor 40×/1.30; Carl Zeiss GmbH). The blue and solid lines show the mTurquoise2 fluorescence intensity over time, the interrupted lines show the sensitized emission level over time. The black graph in a separate upper right window shows the FRET ratio (YFP/CFP) over time. The error is indicated with grey lines and corresponds to 95% confidence interval. A) Shows the FRET graphs for a Gqsensor including a Gβ sequence and mTurquoise2-cpVenus6 as FRETpair, *n*= 13 (and 2 non-responding cells were imaged) B) Shows the FRET graphs for a Gqsensor without a Gβ sequence and mTurquoise2-SYFP2 as FRETpair, *n*=42 (and 2 non-responding cells were imaged).

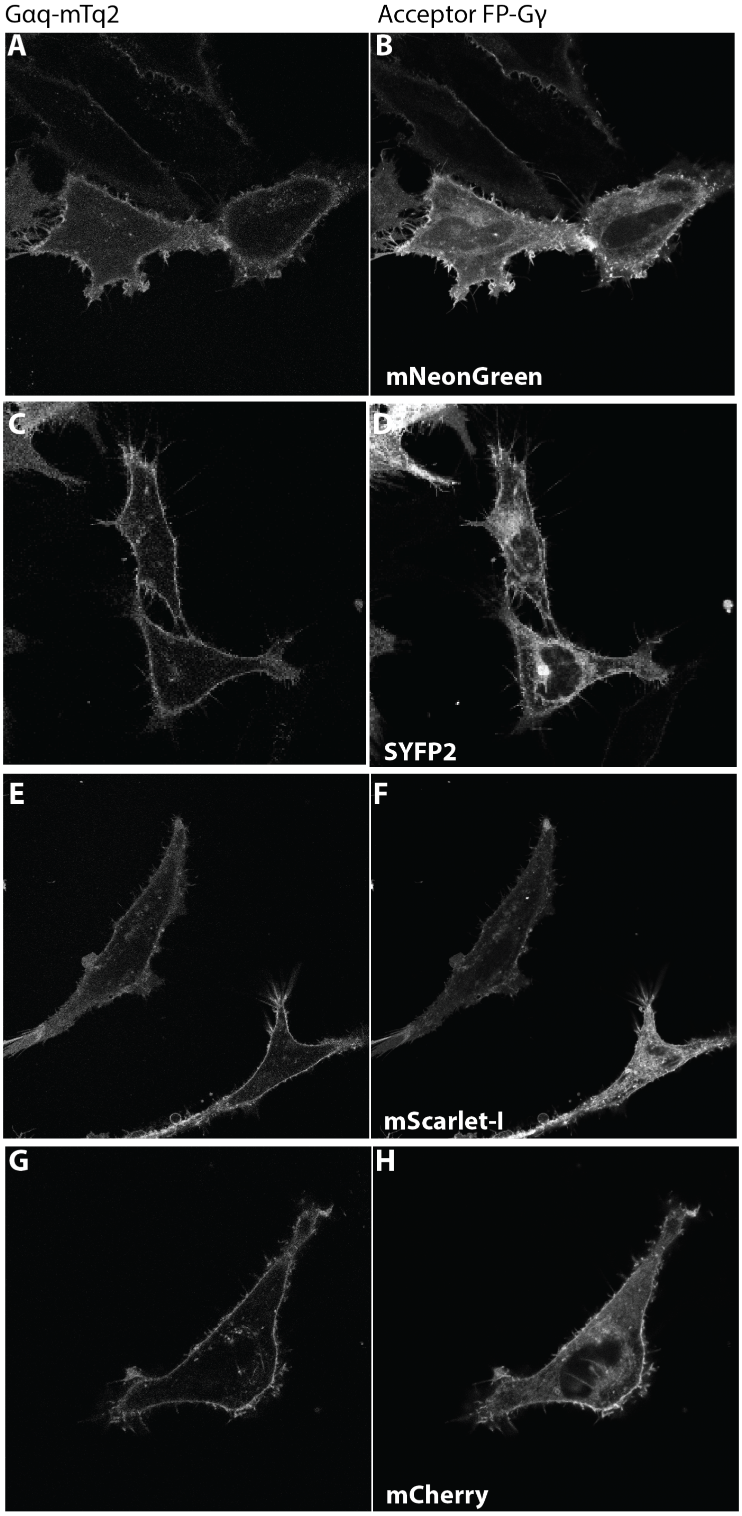

Figure S8. Localization of Gq activation biosensors.
This figure shows images of the localization of the Gq-sensors containing a Gαq-mTurquoise2 (A, C, E, G) and a Gγ tagged with either mNeonGreen (B), SYFP2 (D) mScarlet-I (F) or mCherry (H). Cells were imaged with a Nikon A1 confocal microscope equipped with a 60x oil immersion objective (Plan Apochromat VC, NA 1.4), with the pinhole size set at 1 Airy Unit. For recording the images of mTurquoise2 the 457nm laser and BP480/30 emission filter were used, for mNeonGreen and SYFP2 the 514nm laser and BP537/26 emission filter were used and for mCherry and mScarlet-I the 561nm laser and BP595/50 emission filter was used. All images were recorded sequentially to minimalize the amount of bleedthrough. The width of the images is 131.35μm for Gq-sensor-mTq2-mNeonGreen, 116.28μm for Gq-sensor-mTq2-SYFP2, 98.32μm for Gq-sensor-mTq2-mCherry and 160.23μm for Gq-sensor-mTq2-mScarlet-I.

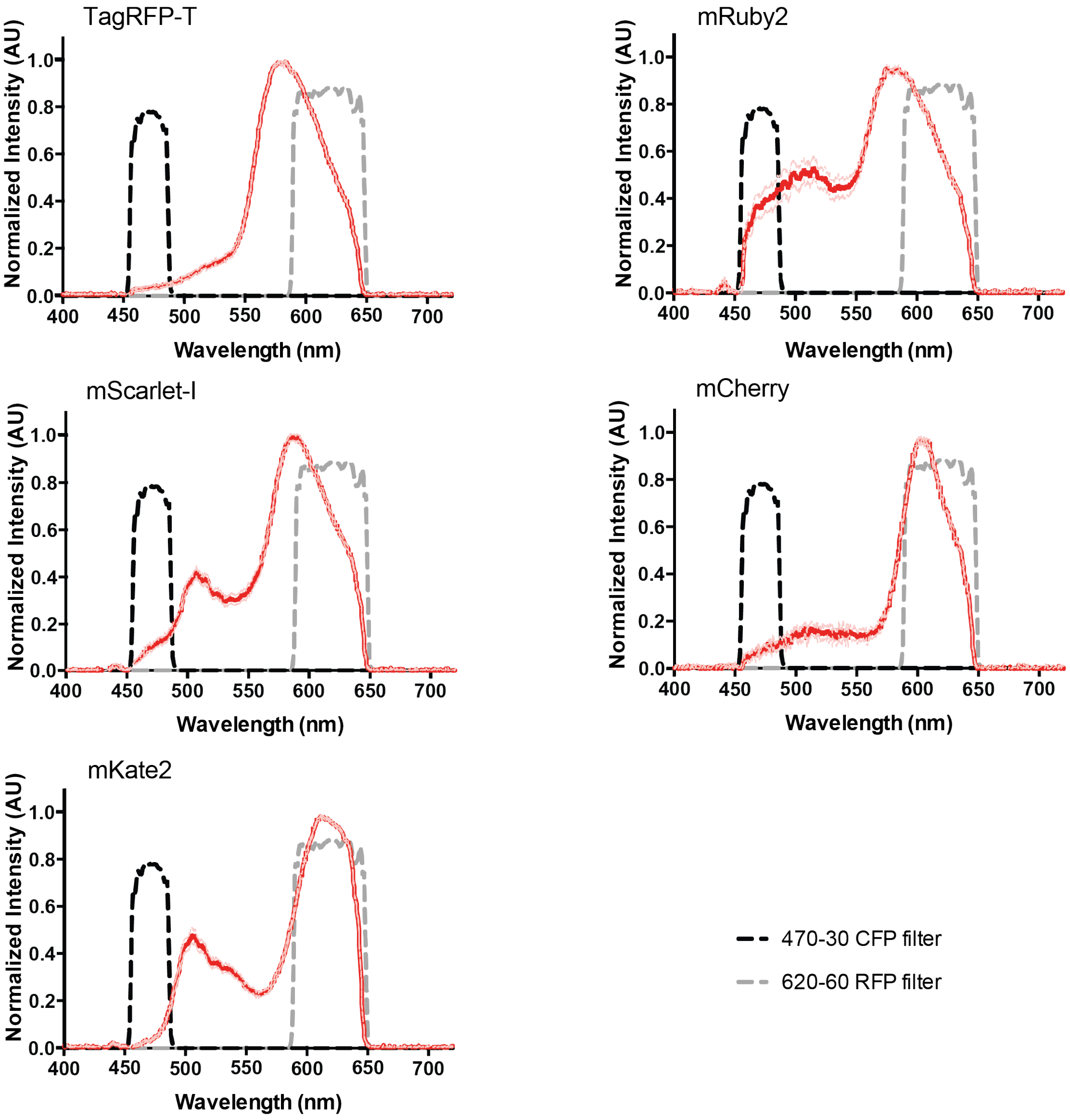

Figure S9. Bleedthrough of RFPs in the CFP emission channel during ratiometric FRET measurements. These graphs show the spectral imaging data recorded for RFPs excited with 436/20nm light, using a 80/20 cube and a 460LP emission filter (red line). The error is indicated with pink lines and corresponds to 95% confidence interval. The emission filters for CFP (BP470-30) (black intermittent line) and RFP (BP620-60) (grey intermittent line) used in ratiometric FRET imaging are shown in the graphs in order to show the bleedthrough of RFPs in the CFP channel and how well certain RFPs fit to the RFP emission filter that we used. Of note, very long exposure times were needed to obtain these spectra since the RFPs are hardly excited with 436/20nm light.   The number of cells analyzed is: TaqRFP-T *n*=15; mRuby2 *n*=16; mScarlet-I *n*=14; mCherry *n*=8; mKate2 *n*=16.

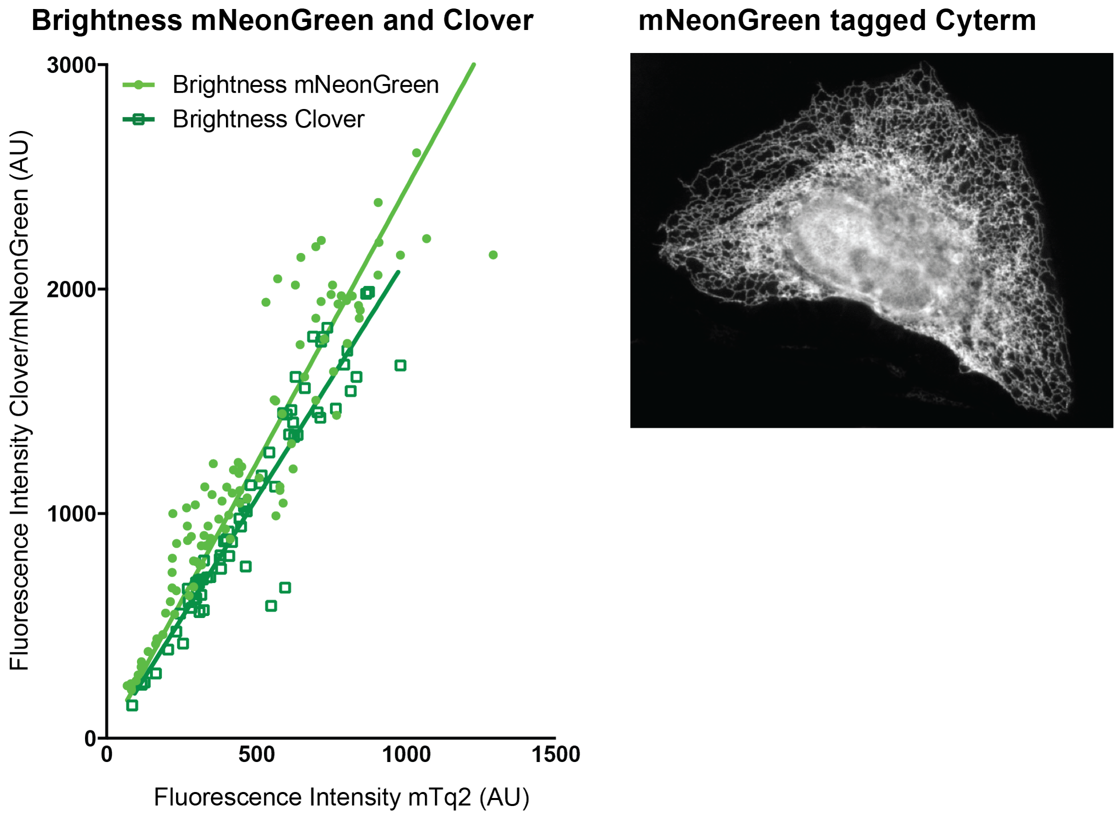

Figure S10. Brightness and dimerization of mNeonGreen. The left graph shows a brightness comparison of mNeonGreen and Clover relative to mTurquoise2 by employing a T2A construct with both FPs (mTurquoise2 and either Clover or mNeonGreen). Cells were imaged on a widefield fluorescence microscope (Axiovert 200 M; Carl Zeiss GmbH) equipped with a xenon arc lamp with monochromator (Cairn Research, Faversham, Kent, UK), using a 40x objective (oil-immersion Plan-Neo- fluor 40×/1.30; Carl Zeiss GmbH). Clover and mNeonGreen are excited with 500nm light and emission was detected with a BP535/30 filter. To prevent cross excitation, reference mTurquoise2 was excited with 405nm light and emission was detected with a BP470/30 filter. After subtraction of background signal, the mean fluorescence intensity of the cells was calculated. Each dot represents a cell. The lines show the fit with a linear regression, forced through the origin, the steeper the line the higher the brightness. The number of cells analyzed are: mNeonGreen-T2A-mTq2 n=99 and Clover-T2A-mTq2 n=69. The right picture shows cellular localization of mNeonGreen tagged cytERM, used in the OSER assay to determine dimerization tendency.

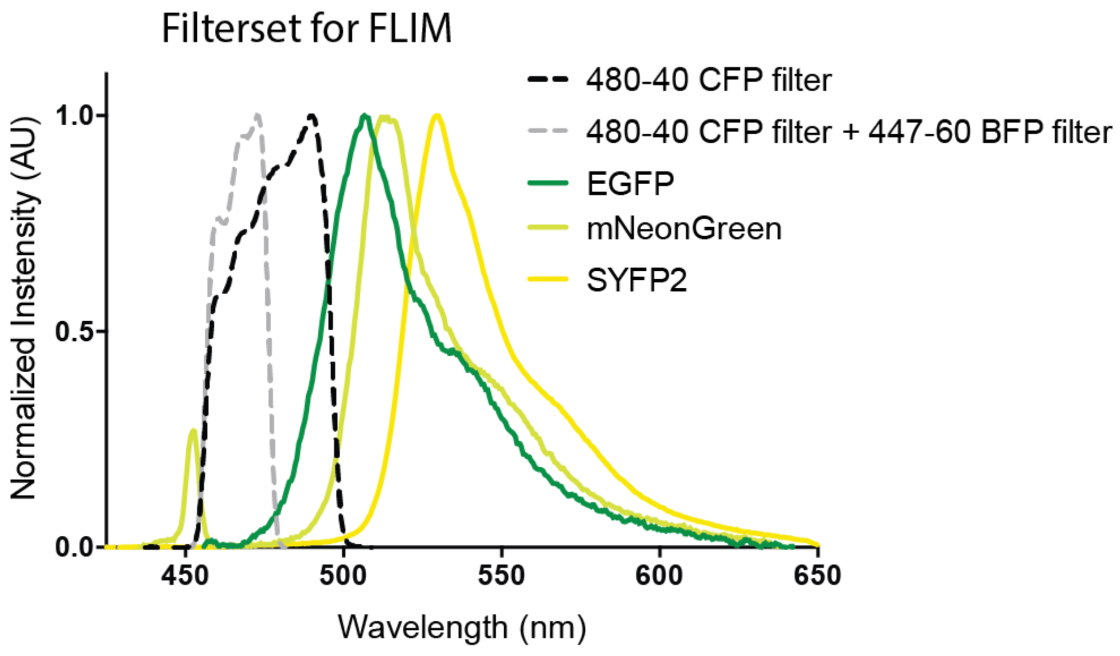

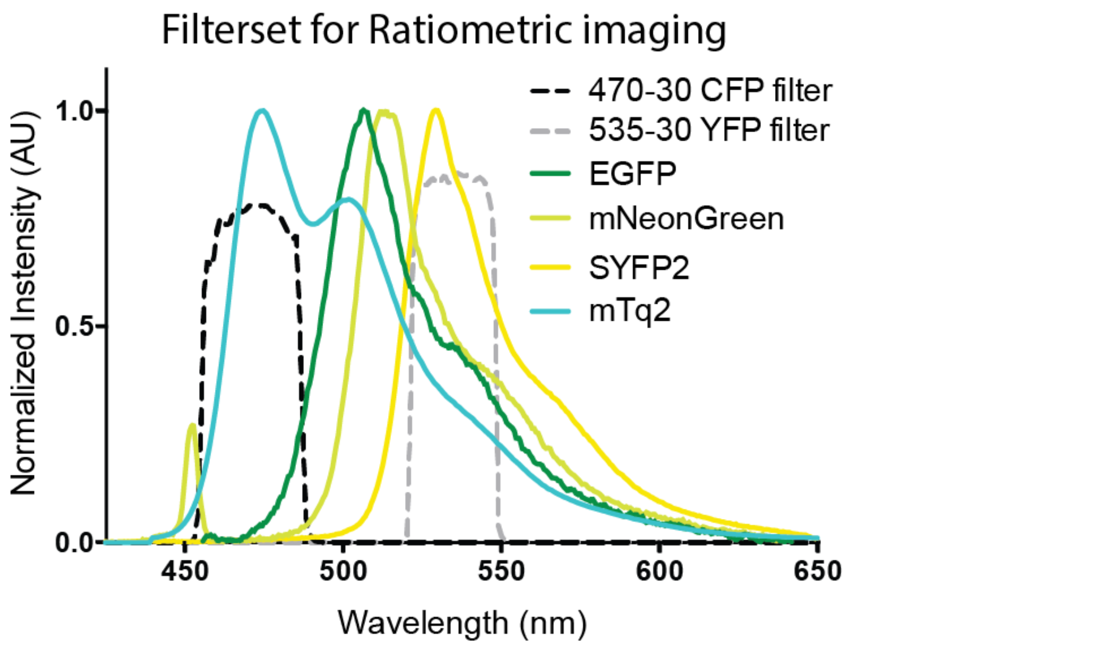

Figure S11. Filtersets for FLIM and Ratiometric FRET imaging.

Graphs depicting emission spectra overlayed with emission bandpass filters. In the graphs of the filterset for FLIM the standard BP480-40 CFP filter (black intermittent line) is compared with the combination of this filter with a BP447-60 BFP filter (grey intermittent line). The figure shows that when using the combination of both filters hardly any emission of mNeonGreen is detected and this filterset is used to record the FLIM data. In the graph of the filterset for ratiometric imaging, the emission filters for CFP (BP470-30) (black intermittent line) and YFP (BP535-30) (grey intermittent line) are shown in combination with the emission spectra of FPs measured in this experiments.

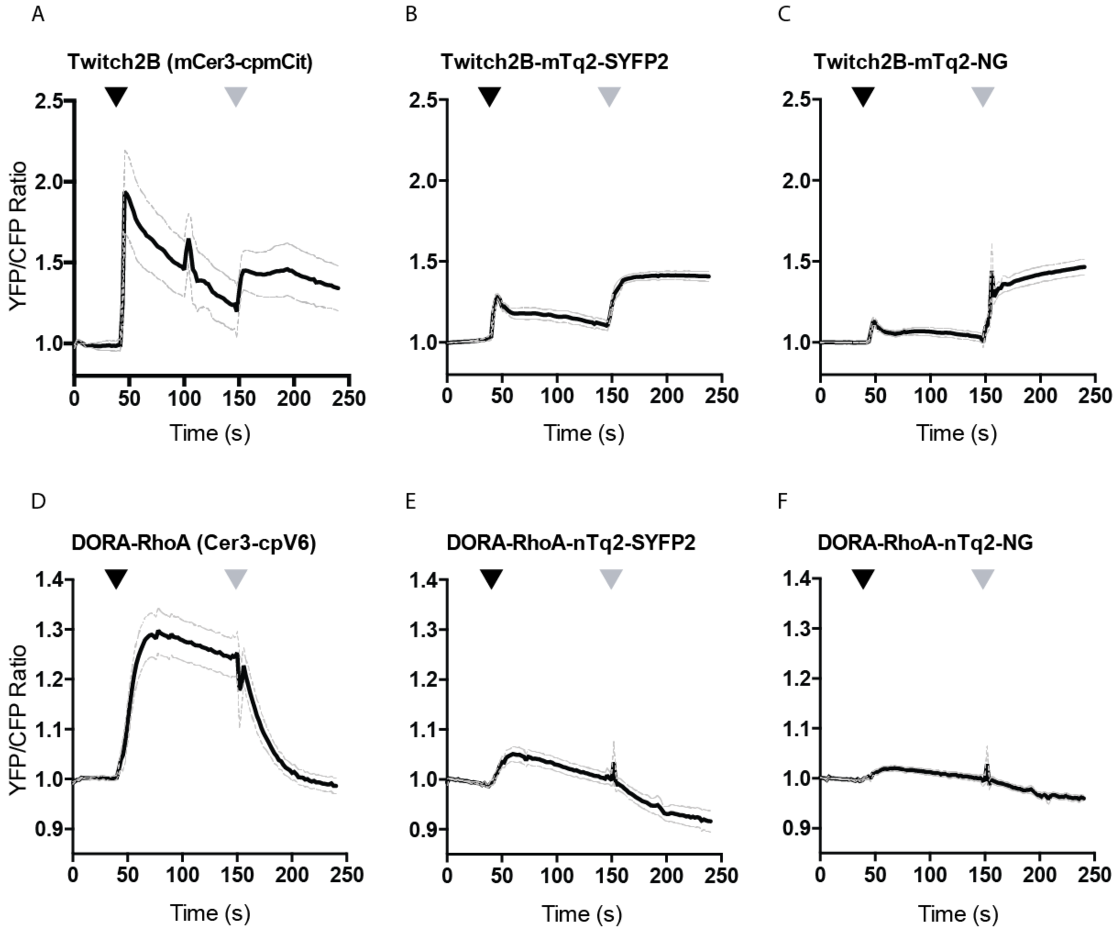

Figure S12. mNeonGreen as acceptor in unimolecular FRET sensors.
These graphs show data of unimolecular sensors for calcium (Twitch2B) (A-C) and RhoA activation (DORA-RHOA) (D-F), comparing the FRET pairs Tq2-SYFP2 (B, E) with Tq2-mNeonGreen (C, F). In the calcium sensor experiments a mTq2 is used, which is Tq2-206K, while in the RhoA activation experiments a nTq2 is used, which is Tq2-206A, because this sensor is believed to rely on stickiness interactions for its high dynamic range. The published, optimized sensors for calcium (A) and RhoA activation (D) are shown, containing respectively mCerulean3 and cpmCitrine, and Cerulean3 and cpVenus6 as FRET pair, showing the original dynamic range of the optimized sensors in which also circular permutations are applied. Cells were imaged with a wide-field fluorescence microscope (Axiovert 200 M; Carl Zeiss GmbH) (Adjobo-Hermans et al., 2011) equipped with a xenon arc lamp with monochromator (Cairn Research, Faversham, Kent, UK) using a 40x objective (oil-immersion Plan-Neo- fluor 40×/1.30; Carl Zeiss GmbH). Fluorophores were excited with 420nm light (slit width 30 nm), mTq2 emission was detected with the BP470/30 filter, GFP/YFP emission was detected with the BP535/30 filter. For the calcium sensor HeLa cells are stimulated with 100µM Histamine at *t*=44s (black arrow) and at *t*= 150s with 10µg/ml Ionomycine (Cayman chemical #10004974) (gray arrow). For the RhoA sensor cells are stimulated with 100µM Histamine at *t*=44s (black arrow) and antagonized at *t*= 150s with 10µM Pyrilamine (gray arrow). In these cells a GEFT-mCherry construct is overexpressed next to the FRET sensor. The error is indicated with grey lines and corresponds to 95% confidence interval. The number of cells imaged is: DORA-RHOA original *n*=26 (and 4 non responding cells were imaged), DORA-RHOA-nTq2-SYFP2 *n*=22, DORA-RHOA-nTq2-mNeonGreen *n*=26, Twitch2B original *n*=19 (and 2 non-responding cells were imaged), Twitch2B-mTq2-SYFP2 *n*=23 and Twitch2B-mTq2-mNeonGreen *n*=26.
